# Association of extracerebral organ failure with 1-year survival and healthcare-associated costs after cardiac arrest: an observational database study

**DOI:** 10.1186/s13054-019-2359-z

**Published:** 2019-02-28

**Authors:** Pirkka T. Pekkarinen, Minna Bäcklund, Ilmar Efendijev, Rahul Raj, Daniel Folger, Erik Litonius, Ruut Laitio, Stepani Bendel, Sanna Hoppu, Tero Ala-Kokko, Matti Reinikainen, Markus B. Skrifvars

**Affiliations:** 1 Division of Intensive Care Medicine, Department of Anaesthesiology, Intensive Care and Pain Medicine, University of Helsinki and Helsinki University Hospital, PB 340, 00029 Helsinki, HUS Finland; 2 Department of Neurosurgery, University of Helsinki and Helsinki University Hospital, Helsinki, Finland; 3 Division of Perioperative Services, Intensive Care and Pain Medicine, Turku University Hospital, Turku, Finland; 4 Division of Intensive Care Medicine, Kuopio University Hospital, Kuopio, Finland; 5 Department of Intensive Care, Tampere University Hospital, Tampere, Finland; 6 Department of Anaesthesiology, University of Oulu, Oulu, Finland; 7 Division of Intensive Care Medicine, Medical Research Center Oulu, Oulu University Hospital, Oulu, Finland; 8 University of Eastern Finland and Kuopio University Hospital, Kuopio, Finland; 9 Department of Emergency Care and Services, University of Helsinki and Helsinki University Hospital, Helsinki, Finland

**Keywords:** Cardiac arrest, OHCA, IHCA, Cost of care, Cost-effectiveness, Organ failure, Multiple organ failure, Outcome, Post cardiac arrest syndrome, SOFA

## Abstract

**Background:**

Organ dysfunction is common after cardiac arrest and associated with worse short-term outcome, but its impact on long-term outcome and treatment costs is unknown.

**Methods:**

We used nationwide registry data from the intensive care units (ICU) of the five Finnish university hospitals to evaluate the association of 24-h extracerebral Sequential Organ Failure Assessment (24h-EC-SOFA) score with 1-year survival and healthcare-associated costs after cardiac arrest. We included adult cardiac arrest patients treated in the participating ICUs between January 1, 2003, and December 31, 2013. We acquired the confirmed date of death from the Finnish Population Register Centre database and gross 1-year healthcare-associated costs from the hospital billing records and the database of the Finnish Social Insurance Institution.

**Results:**

A total of 5814 patients were included in the study, and 2401 were alive 1 year after cardiac arrest. Median (interquartile range (IQR)) 24h-EC-SOFA score was 6 (5–8) in 1-year survivors and 7 (5–10) in non-survivors. In multivariate regression analysis, adjusting for age and prior independency in self-care, the 24h-EC-SOFA score had an odds ratio (OR) of 1.16 (95% confidence interval (CI) 1.14–1.18) per point for 1-year mortality.

Median (IQR) healthcare-associated costs in the year after cardiac arrest were €47,000 (€28,000–75,000) in 1-year survivors and €12,000 (€6600–25,000) in non-survivors. In a multivariate linear regression model adjusting for age and prior independency in self-care, an increase of one point in the 24h-EC-SOFA score was associated with an increase of €170 (95% CI €150–190) in the cost per day alive in the year after cardiac arrest. In the same model, an increase of one point in the 24h-EC-SOFA score was associated with an increase of €4400 (95% CI €3300–5500) in the total healthcare-associated costs in 1-year survivors.

**Conclusions:**

Extracerebral organ dysfunction is associated with long-term outcome and gross healthcare-associated costs of ICU-treated cardiac arrest patients. It should be considered when assessing interventions to improve outcomes and optimize the use of resources in these patients.

**Electronic supplementary material:**

The online version of this article (10.1186/s13054-019-2359-z) contains supplementary material, which is available to authorized users.

## Background

Cardiac arrest is an extensively studied topic due to its devastating consequences [1]. Cardiac arrest may itself cause end-organ damage leading to life-threatening organ dysfunction despite successful return of spontaneous circulation (ROSC) [2]. In addition to this the systemic inflammatory response which follows (often called “post cardiac arrest syndrome”) is characterized by a sepsis-like inflammatory response which may precipitate multiple organ failure (MOF) and death [3, 4]. Contemporary treatment of cardiac arrest patients in the ICU is focused on supporting the recovery of the central nervous system and the myocardium after ischaemia. The dysfunction of other organ systems after cardiac arrest may have an impact on recovery but has received less attention.

The Sequential (or sepsis-related) Organ Failure Assessment (SOFA) score is widely used for estimating the severity of MOF. The SOFA score was originally developed for the evaluation of organ failure in sepsis [5], but it has later been validated as a general scoring system for all critically ill patients [6–11].

The extracerebral (EC-)SOFA score [i.e. SOFA score excluding the central nervous system (CNS) sub-score] can be used to evaluate the degree of MOF outside the central nervous system. Two previous single-centre studies have shown an association of EC-SOFA with in-hospital and 28-day mortality in cardiac arrest patients [12, 13], and one multi-centre study found an association of admission renal SOFA sub-score with ICU mortality [14].

The aim of this study was to quantify the impact of early extracerebral organ failure, measured as the EC-SOFA score at 24 h (24h-EC-SOFA), on 1-year survival and healthcare-associated costs in cardiac arrest patients treated in the ICU.

## Methods

### Study population

This is a retrospective study conducted at the five university hospitals of Finland. These five centres serve a population of 3.2 million covering 60% of the entire population of Finland. We included adult cardiac arrest patients treated in the participating ICUs between January 1, 2003, and December 31, 2013. The year 2003 was chosen as the starting point because it was the first year when therapeutic hypothermia became widely used in the treatment of cardiac arrest patients in the participating centres [15]. Only the first event of cardiac arrest was considered. Cardiac arrest was recognized as admission diagnosis of cardiac arrest according to Acute Physiology and Chronic Health Evaluation III (APACHE III) or positive value for Therapeutic Intervention Scoring System 76 (TISS-76) item of “cardiac arrest and/or countershock within past 48 h”. Complete data for admission date and day of death/vital status at the end of 1-year follow-up was required for inclusion. Patients with missing SOFA score were excluded.

### Database

The Finnish social security and healthcare system is government-based. Each Finnish resident is assigned a unique personal identification code which is used to identify the person in medical records, social security records, and national registers. We used this identification code to combine data from different sources.

Medical data were extracted from the Finnish Intensive Care Consortium (FICC) database [16]. These included the SOFA score and the premorbid physical status as a simplified World Health Organization-Eastern Cooperative Oncology Group (WHO/ECOG) classification [17], where “independent” was defined as the patient being independent in self-care and “dependent” was defined as the patient being partly or fully dependent on help in self-care prior to hospital admission. We linked these data with the confirmed date of death obtained from the Finnish Population Register Centre database, which records all deaths of Finnish residents.

Furthermore, we linked data on gross 1-year healthcare-associated costs from the hospital billing records, rehabilitation costs and social security costs obtained from the Social Insurance Institution. The hospital costs include all costs until hospital discharge (diagnostics, ICU stay, ward stay, operative treatment etc.). The rehabilitation costs were calculated by multiplying the number of days spent in the rehabilitation unit with the average price per day for units of the corresponding level. The Finnish Social Insurance Institution reimburses disability and sickness allowances, private physician and physiotherapist expenses, prescription drug expenses and medical transportation expenses. All social insurance reimbursements during the year following hospital admission were included. Effective cost per survivor (ECPS) was calculated by dividing the sum of total costs for all patients with the number of survivors [18]. We adjusted all costs to the value of euro in the year 2013 according to the consumer price index (CPI) in Finland.

### Nested cohort

We performed an additional analysis of a nested cohort consisting of patients treated in one (Helsinki University Hospital) of the five centres of the full data. For this cohort, additional data were available according to the Utstein criteria [19, 20] including the delay from collapse to return of spontaneous circulation (ROSC), type of initial rhythm during cardiac arrest (shockable or not), whether the cardiac arrest was witnessed or not, and 1-year cerebral performance category (CPC) [21, 22]. We defined good neurologic outcome as 1-year CPC 1 or 2 and poor neurologic outcome as 1-year CPC 3 to 5. Furthermore, the location where cardiac arrest had occurred was recorded, allowing stratification of the nested cohort data in out-of-hospital cardiac arrest (OHCA) and in-hospital cardiac arrest (IHCA). Cardiac arrests in the ICU (ICUCA) were included in the IHCA group in this study.

### Statistics

We performed the statistical analyses with the SPSS software (version 24.0, IBM, Armonk, NY, USA). We present continuous data as medians with interquartile ranges (IQR) and categorical data as percentages of the whole. We tested group differences with a chi-square test, Student’s *t* test, Mann-Whitney *U* test or Kruskal-Wallis test, as appropriate. We performed Kaplan-Meier analysis to evaluate the difference in 1-year survival across quartiles of the 24h-EC-SOFA score. We used logistic regression models to search for variables that were independently associated with outcome. For testing the association of the 24h-EC-SOFA score with healthcare-associated costs, we used linear regression models. To avoid the confounding effect of early deaths causing low costs, we calculated a “cost per day alive” variable by dividing the total costs of a given patient by the number of days the patient remained alive during the first year after the cardiac arrest. If the patient was still alive 1 year after the arrest, then the cost per day alive variable was calculated by dividing the total costs during the year after cardiac arrest by 365.

### STROBE statement

The manuscript was completed according to STROBE standards [23].

## Results

**Fig. 1:**
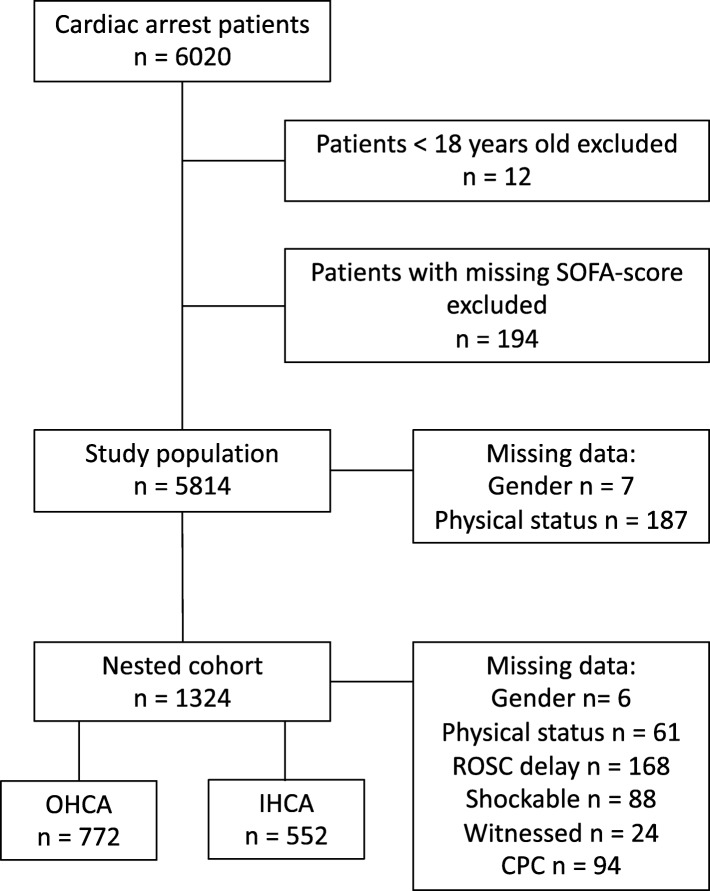
Flow chart of patient selection

In total, 5814 patients were included in the study, and of these, 2401 were alive 1 year after the cardiac arrest. Median (IQR) survival time for 1-year non-survivors was 5 (2–14) days. Patient selection is shown in Fig. 1. Characteristics of the study population are described in Table 1, and those of the nested cohort in Additional file 1: Table S1. The values of the 24h-EC-SOFA score were normally distributed in the groups stratified by outcome, with a range from 0 to 18 (Additional file 2: Figure S1). The distribution of the full 24h-SOFA score ranged from 0 to 21 (Additional file 2: Figure S1).

**Table 1:** Characteristics of the study population

	All *N* = 5814 (100%)	One-year survivors *N* = 2401 (41%)	One-year non-survivors *N* = 3413 (59%)	*P* value^1^
Age (years)	65 (56–74)	63 (54–71)	67 (57–76)	< 0.01
Gender (male)	71%	72%	69%	< 0.01
Physical status (independent)^2^	84%	92%	79%	< 0.01
24h-EC-SOFA	7 (5–9)	6 (5–8)	7 (5–10)	< 0.01
24 h-SOFA	9 (7–12)	8 (6–10)	10 (8–13)	< 0.01
One-year costs (€1000)	23 (10–50)	47 (28–75)	12 (6.6–25)	< 0.01
Cost per day alive (€)	880 (150–2500)	130 (77–200)	2200 (1300–3400)	< 0.01

^1^*P* value for comparison between outcome groups

^2^Simplified WHO/ECOG-classification before cardiac arrest

### Outcome

**Fig. 2:**
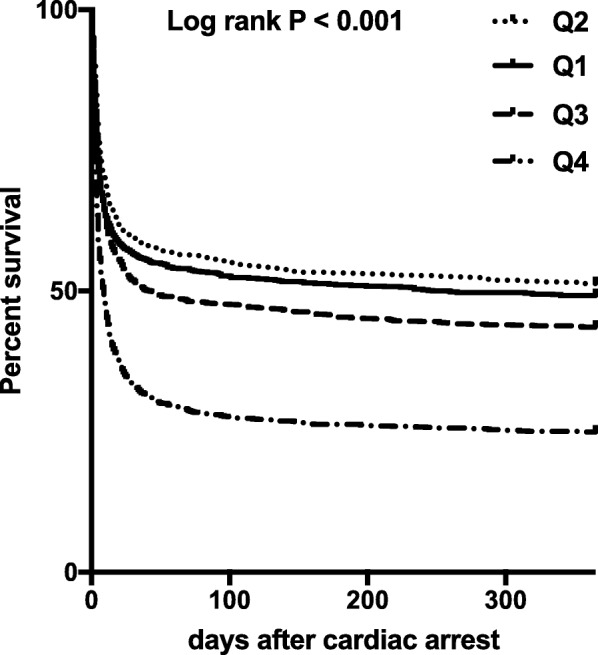
Kaplan-Meier curves for survival up to 1 year after cardiac arrest in quartiles of the EC-SOFA score. Q1 quartile 1, EC-SOFA score 0–4; Q2, 5–6; Q3, 7–8; Q4, 9–18. Logrank *P* value for equality of survival distributions across quartiles of EC-SOFA

In Kaplan-Meier analysis, 1-year survival was significantly different between quartiles of 24h-EC-SOFA (logrank *P* < 0.001; Fig. 2). One-year survival had a rising trend from 38% in 2003 to 47% in 2013 (Additional file 3: Figure S2). In a logistic regression model adjusting for age and the premorbid physical status, the 24h-EC-SOFA score was associated with 1-year mortality [odds ratio (OR) 1.16 and 95% confidence interval (CI) 1.14–1.18; Table 2]. Similarly, the full 24h-SOFA score was associated with 1-year mortality in a logistic regression model adjusting for age and the premorbid physical status (OR 1.22, 95% CI 1.20–1.24; Additional file 4: Table S2). All sub-scores, except the cardiovascular sub-score, were independently associated with 1-year mortality when the individual EC-sub-scores were tested (respiration, OR 1.18, 95% CI 1.12–1.25; coagulation, OR 1.09, 95% CI 1.00–1.18; liver, OR 1.23, 95% CI 1.10–1.37; renal, OR 1.52, 95% CI 1.44–1.61; Additional file 5: Table S3). Univariate analysis of the variables used in the logistic regression models is presented in Additional file 6: Table S4.

**Table 2:** Logistic regression model of the association of the 24h-EC-SOFA score with 1-year outcome

	Full data			Nested cohort					
	One-year mortality			One-year mortality			Poor neurologic outcome^1^		
	OR	95% CI	*P*	OR	95% CI	*P*	OR	95% CI	*P*
Age (year)	1.02	1.01 - 1.02	< 0.01	1.03	1.02 - 1.04	< 0.01	1.03	1.02 - 1.04	< 0.01
Physical status (dependent)^2^	2.60	2.18 - 3.09	< 0.01	2.76	1.60 - 4.74	< 0.01	3.72	2.01 - 6.88	< 0.01
Not shockable^3^				3.55	2.61 - 4.82	< 0.01	3.89	2.82 - 5.37	< 0.01
ROSC delay (min)^4^				1.05	1.03 - 1.06	< 0.01	1.05	1.03 - 1.06	< 0.01
Not witnessed^5^				1.87	1.19 - 2.93	< 0.01	2.05	1.26 - 3.32	< 0.01
24h-EC-SOFA (point)	1.16	1.14 - 1.18	< 0.01	1.19	1.12 - 1.25	< 0.01	1.15	1.09 - 1.21	< 0.01

^1^Cerebral performance category (CPC) 3–5 1 year after cardiac arrest

^2^Simplified WHO/ECOG-classification before cardiac arrest

^3^Not shockable, initial cardiac rhythm during resuscitation not shockable (asystole/pulseless electrical activity)

^4^ROSC delay, time from collapse to return of spontaneous circulation

^5^Not witnessed, collapse not witnessed

### Healthcare-associated costs

**Fig. 3:**
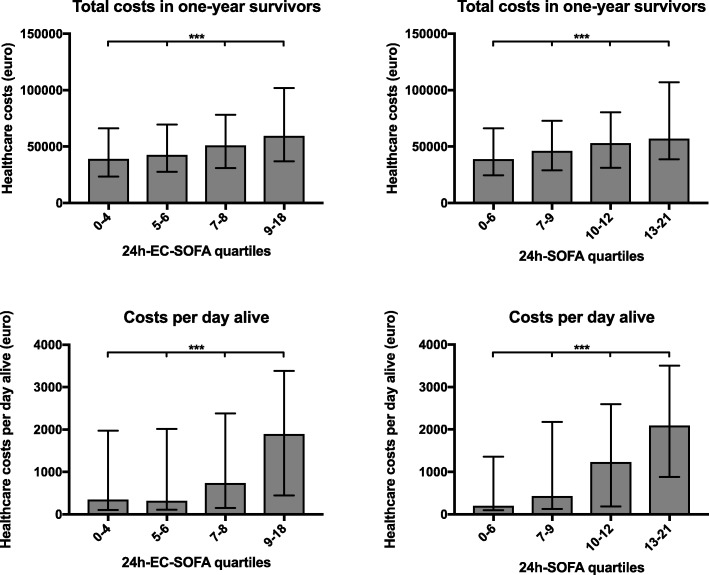
Healthcare-associated costs recorded during the first year after cardiac arrest in quartiles of 24h-EC-SOFA and 24h-SOFA scores. Upper panels: Total healthcare-associated costs in 1-year survivors in quartiles of 24h-EC-SOFA score (left) and full 24h-SOFA score including CNS sub-score (right). Lower panels: healthcare-associated costs per day alive in quartiles of the 24h-EC-SOFA score (left) and full 24h-SOFA score including CNS sub-score (right). The cost per day alive was calculated by dividing the total cost by the number of days from cardiac arrest to death. For 1-year survivors, the cost per day alive was calculated by dividing the total cost by 365 days. Median values (bars) with interquartile range (whiskers) are presented. ***P < 0.001 for the difference between the four groups (Kruskal-Wallis test)

The sum of costs recorded in all 5814 patients during the year following cardiac arrest was €230,000,000. The 1-year ECPS was €96,000. Median costs in the first year after cardiac arrest were €47,000 in 1-year survivors and €12,000 in non-survivors (Table 1). During the study years, ECPS remained between €85,000 and €110,000 (Additional file 7: Figure S3). Median costs recorded for 1-year survivors were lowest in 2003 (€38,000) and highest in 2004 (€52,000), plateauing thereafter (Additional file 8: Figure S4). Median costs recorded for 1-year non-survivors had a rising trend from €9900 in 2003 to €16,000 in 2013(Additional file 8: Figure S4). When the data were divided in quartiles of the 24h-EC-SOFA score, the total costs of 1-year survivors differed significantly between groups (*P* <  0.001); higher 24h-EC-SOFA score was associated with higher costs (Fig. 3). The same was true when the quartiles of the 24h-EC-SOFA score were tested for the association with cost per day alive during the first year after cardiac arrest (*P* < 0.001). The differences were similar for quartiles of the 24 h-SOFA score (*P* < 0.001 for the difference in total costs in 1-year survivors and *P* < 0.001 for the difference in cost per day alive; Fig. 3).

In a linear regression model adjusting for age and premorbid physical status, an increase of one point in the 24h-EC-SOFA score was associated with an increase of €170 (95% CI €150–190) in the cost per day alive in the first year after cardiac arrest (Table 3). When the EC-sub-scores were tested, renal, cardiovascular, respiratory and coagulation sub-scores were significantly associated with an increase in the cost per day alive (increase per one point rise: respiration, €120, 95% CI €61 - 170; coagulation, €300, 95% CI €230 - 370; cardiovascular, €90, 95% CI €41 - 140; renal, €400, 95% CI €350 – 440; Additional file 9: Table S5). In 1-year survivors, an increase of one point in the 24h-EC-SOFA score was associated with an increase of €4400 (95% CI €3300–5500) in total healthcare-associated costs recorded during the year following the cardiac arrest (Table 4). When the EC-sub-scores were tested, renal, respiratory and coagulation sub-scores were significantly associated with an increase of total healthcare-associated costs recorded during the year following the cardiac arrest (increase per one point rise: respiration, €7300, 95% CI €4800 - 9800; coagulation, €4600, 95% CI €1000 - 8300; renal, €7900, 95% CI €5000 - 11,000; Additional file 10: Table S6).

**Table 3:** Linear model of the association of the 24h-EC-SOFA score with 1-year healthcare-associated costs per day alive

	Cost per day alive (€)					
	Full data			Nested cohort		
	*B*	95% CI	*P*	*B*	95% CI	*P*
Age (year)	− 3.8	− 8.4 - 0.80	NS	3.8	− 5.1 - 13	NS
Physical status (dependent)^1^	110	− 67 - 284	NS	220	− 240 - 680	NS
Not shockable^2^				780	510 - 1,100	< 0.01
ROSC (min)^3^				37	25 - 49	< 0.01
Not witnessed^4^				200	− 200 - 610	NS
24h-EC-SOFA (point)	170	150 - 190	< 0.01	210	170 - 260	< 0.01

*NS* not statistically significant (*P* > 0.05)

^1^Simplified WHO/ECOG-classification before cardiac arrest

^2^Not shockable, initial cardiac rhythm during resuscitation not shockable (asystole/pulseless electrical activity)

^3^ROSC delay, time from collapse to return of spontaneous circulation

^4^Not witnessed, collapse not witnessed

**Table 4:** Linear model of the association of the 24h-EC-SOFA score with 1-year total healthcare-associated costs (per €1000) in 1-year survivors

	Total costs in 1-year survivors (€1000)					
	Full data			Nested cohort		
	*B*	95% CI	*P*	*B*	95% CI	*P*
Age (year)	− 0.85	− 1.1 - -0.64	< 0.01	− 0.48	− 0.85 - –0.10	0.01
Physical status (dependent)^1^	15	5.1 - 25	< 0.01	28	2.4 - 54	0.03
Not shockable^2^				15	3.2 - 27	0.01
ROSC (min)^3^				0.047	− 0.45 - 0.55	NS
Not witnessed^4^				− 20	− 39 - -0.91	0.04
24h-EC-SOFA (point)	4.4	3.3 - 5.5	< 0.01	7.2	5.0 - 9.4	< 0.01

*NS* not statistically significant (*P* > 0.05)

^1^Simplified WHO/ECOG-classification before cardiac arrest

^2^Not shockable, initial cardiac rhythm during resuscitation not shockable (asystole/pulseless electrical activity)

^3^ROSC delay, time from collapse to return of spontaneous circulation

^4^Not witnessed, collapse not witnessed

### Outcome in the nested cohort

For the nested cohort, the logistic regression model was complemented with variables describing conditions during resuscitation: whether the initial cardiac rhythm during cardiac arrest was shockable, the delay from collapse to ROSC and whether the cardiac arrest was witnessed. In this model, the 24h-EC-SOFA score was associated with 1-year mortality (OR 1.19, 95% CI 1.12–1.25 per point of 24h-EC-SOFA) and poor neurologic outcome (OR 1.15, 95% CI 1.09–1.21 per point of 24h-EC-SOFA; Table 2). When the individual EC-sub-scores were tested, respiratory and renal sub-scores were independently associated with both 1-year mortality (respiration, OR 1.16, 95% CI 1.02–1.32; renal, OR 1.50, 95% CI 1.32–1.70) and poor neurologic outcome (respiration, OR 1.17, 95% CI 1.02–1.33; renal, OR 1.42, 95% CI 1.25–1.63; Additional file 11: Table S7). The association of the 24h-EC-SOFA score with 1-year mortality and poor neurologic outcome remained significant in subgroup analysis of both OHCA (1-year mortality OR 1.20, 95% CI 1.10–1.31 per point of 24h-EC-SOFA; poor neurologic outcome OR 1.15, 95% CI 1.05–1.25 per point of 24h-EC-SOFA) and IHCA patients (1-year mortality OR 1.15, 95% CI 1.06–1.24 per point of 24h-EC-SOFA; poor neurologic outcome OR 1.11, 95% CI 1.03–1.21 per point of 24h-EC-SOFA; Additional file 12: Table S8).

### Healthcare-associated costs in the nested cohort

In the nested cohort, the total costs documented for the 1324 patients was €65,000,000 and 1-year ECPS was €94,000. The cohort included 772 OHCA and 552 IHCA patients. Median (IQR) survival time for 1-year non-survivors was 5 (3–10) days in OHCA and 8 (3–20) days in IHCA. In the nested cohort, the linear regression model adjusting for age and premorbid physical status indicated an association of an increase of one point in the 24h-EC-SOFA score with a €280 (95% CI €230–330) increase in the cost per day alive. For 1-year survivors, the increase in the total healthcare-associated costs in the year after cardiac arrest was €7700 (95% CI €5700–9700) per one-point rise in the 24h-EC-SOFA score. When the model was complemented with Utstein variables, the increase in the cost per day alive was €210 (95% CI €170–260) per 24h-EC-SOFA point. In 1-year survivors, total healthcare-associated costs increased €7200 (95% CI €5000–9400) per each additional 24h-EC-SOFA point (Tables 3 and 4). When the data were stratified according to cardiac arrest location, the increase in the cost per day alive in OHCA was €210 (95% CI €150–280) and in IHCA €200 (95% CI €120–270) per 24h-EC-SOFA point. In OHCA patients, the total healthcare-associated costs in 1-year survivors increased €2500 (95% CI €200–4800) and in IHCA patients €11,000 (95% CI €5600–16,000) per each additional 24h-EC-SOFA point (Additional files 13 and 14: Tables S9 and S10).

## Discussion

In this large multi-centre cohort, we show that extracerebral organ failure is an important contributor to both long-term outcome and increased healthcare-associated costs in cardiac arrest patients. Importantly, we measured extracerebral organ failure with a simple method, the 24h-EC-SOFA, and were able to quantify its association with long-term resource use in cardiac arrest survivors.

### Outcome and SOFA

Previous studies have shown an association of admission SOFA and EC-SOFA with 28-/30-day mortality in single-centre settings of OHCA patients [13, 24, 25]. Association with neurologic outcome has produced conflicting results [24, 25]. In a single-centre material including both OHCA and IHCA patients, the highest EC-SOFA score during the first 72 h of ICU stay was associated with in-hospital mortality and cardiovascular and respiratory sub-scores were independently predictive of in-hospital mortality [12]. In a multi-centre database study including both OHCA and IHCA patients, ICU non-survivors had higher cardiovascular, respiratory and renal SOFA sub-scores upon ICU admission but only renal sub-score was independently associated with ICU-mortality [14].

To the best of our knowledge, our current study is the largest study on the association of EC-SOFA with cardiac arrest patient outcome published to date. In our multi-centre setting with a mixed cohort of OHCA and ICHA patients, the association of EC-SOFA with 1-year outcome was clear. In contrast to other sub-scores, the cardiovascular sub-score was not independently associated with the outcome in our cohort, since almost three quarters of patients had 24-h cardiovascular sub-score of 3 or 4, regardless of the outcome group. In line with previous studies, respiratory and renal sub-scores had a clear association with the outcome in our study population.

It is important to notice that the large overlap between the outcome groups does not allow the use of 24h-EC-SOFA for predicting outcome on a single patient level. However, our results emphasize the importance of the evolving multiple organ failure for long-term morbidity, mortality and healthcare resource use in cardiac arrest patients on population level. This association could justify the use of SOFA or EC-SOFA score as an end-point in small randomized controlled trials on cardiac arrest in the future instead of mortality, for which the studies often prove underpowered.

### Healthcare-associated costs

Recently, there has been a growing interest in the obvious socioeconomic burden of cardiac arrest [26]. One of the first studies assessing the healthcare resource use in cardiac arrest reported combined pre- and in-hospital costs for OHCA 6-month survivors to be €36,000 (expressed as 2013 euros) [27]. More recent studies have reported in-hospital costs of €50,000–60,000 in 2013 euros for patients surviving to hospital discharge after OHCA or IHCA [28–30]. One previous study reported OHCA survivors to have combined pre-, in- and post-hospital healthcare-associated costs of €37,000 in 2013 euros in a 6-month follow-up [31]. In a recent study of single-centre data published by our group, combined in- and post-hospital healthcare costs in 1-year follow-up were €59,000 for OHCA and €84,000 for IHCA hospital survivors in 2013 euros [18].

In our current study, the association of 24h-EC-SOFA with healthcare-associated costs was clear. Most of the sub-scores (renal and respiratory sub-scores in particular) had also independent association with costs. This suggests that early organ failure after cardiac arrest leads to both short- and long-term morbidity reflected as increased in- and post-hospital expenses.

The analysis of our nested cohort clearly shows that the association of organ failure with outcome and costs is not redundant to more detailed information considering the conditions during and after cardiac arrest and resuscitation. The similarity of the ECPS values of the nested cohort and the full data (€94,000 and €96,000, respectively) suggest that the case-mix in the nested cohort was not markedly different from that of the full data.

### Strengths and limitations of the study

This study has a number of strengths. It is a large multi-centre study conducted in a country of government-funded social security and healthcare systems. The follow-up time of 1 year is long enough to reliably dissect long-term survivors from non-survivors. We have exploited the unique possibility provided by the nature of the government-funded system to combine data from different registries to acquire information on long-term survival and gross healthcare-associated costs from different healthcare providers. Naturally, there are also limitations. Our data are limited to one country—the generalizability to other healthcare systems is not certain. The study is retrospective, and the data were gathered during a period of 11 years. The treatment of cardiac arrest patients has evolved during the data gathering period and the 4-year delay from the end of follow-up to the publication of the study. Unfortunately, distinction between OHCA and IHCA, which have largely different aetiology, was not possible in the main cohort. Furthermore, the FICC database provided only a point measure of SOFA score as the worst values recorded during the first 24 h, not allowing for evaluation of the temporal change in SOFA score.

## Conclusions

We conclude that extracerebral organ failure, measured as the 24h-EC-SOFA score, is associated with long-term outcome and gross healthcare-associated costs of ICU-treated cardiac arrest patients. These associations encourage future research on the potential of EC-SOFA or SOFA score as end points in randomized controlled trials on cardiac arrest. From the clinical point of view, our data remind us that the full spectrum of multiple organ failure must be considered in the pursuit to reduce morbidity, increase survival, and optimize the use of healthcare resources after cardiac arrest.

## Additional files

Additional file 1: **Table S1.** Characteristics of the nested cohort. (PDF 51 kb)

Additional file 2: **Figure S1.** Distribution of EC-SOFA and SOFA score in outcome groups. (PDF 55 kb)

Additional file 3: **Figure S2.** One-year survival stratified by admission year. (PDF 38 kb)

Additional file 4: **Table S2.** Logistic regression model for the association of the SOFA score with outcome. (PDF 43 kb)

Additional file 5: **Table S3.** Logistic regression model for the association of the EC-SOFA sub-score with outcome in the full data. (PDF 40 kb)

Additional file 6: **Table S4.** Univariate analysis of variables used in multivariate models. (PDF 69 kb)

Additional file 7: **Figure S3.** ECPS stratified by admission year. (PDF 40 kb)

Additional file 8: **Figure S4.** Total costs per patient stratified by admission year. (PDF 46 kb)

Additional file 9: **Table S5.** Logistic regression model for the association of the EC-SOFA sub-score with outcome in the nested cohort. (PDF 40 kb)

Additional file 10: **Table S6.** Logistic regression model for the association of the EC-SOFA score with outcome in OHCA and IHCA sub-groups of the nested cohort. (PDF 40 kb)

Additional file 11: **Table S7.** Linear regression model for the association of the EC-SOFA sub-score with cost per day alive. (PDF 43 kb)

Additional file 12: **Table S8.** Linear regression model for the association of the EC-SOFA sub-score with total costs in 1-year survivors. (PDF 46 kb)

Additional file 13: **Table S9.** Linear regression model for the association of the EC-SOFA sub-score with cost per day alive in OHCA and IHCA sub-groups of the nested cohort. (PDF 43 kb)

Additional file 14: **Table S10.** Linear regression model for the association of the EC-SOFA sub-score with total costs in 1-year survivors in OHCA and IHCA sub-groups of the nested cohort. (PDF 43 kb)

## Abbreviations

24h-EC-SOFA: Extracerebral sequential organ-failure assessment, worst values of the first 24 h
CI: Confidence interval
CNS: Central nervous system
CPC: Cerebral Performance Categories
CPI: Consumer price index
ECPS: Effective cost per survivor
EC-SOFA: Extracerebral Sequential Organ Failure Assessment (excluding the CNS sub-score)
FICC: Finnish Intensive Care Consortium
ICU: Intensive care unit
IHCA: In-hospital cardiac arrest
IQR: Inter-quartile range
MOF: Multiple organ failure
OHCA: Out-of-hospital cardiac arrest
OR: Odds ratio
ROSC: Return of spontaneous circulation
SOFA: Sequential Organ Failure Assessment
WHO/ECOG: World Health Organization-Eastern Cooperative Oncology Group
