# Supplementary material for: Association of extracerebral organ failure with 1-year survival and healthcare-associated costs after cardiac arrest: an observational database study
Source: Crit Care. 2019 Feb 28;23:67. doi: 10.1186/s13054-019-2359-z (PMC6396453; doi:10.1186/s13054-019-2359-z)
Supplement: Supplementary file 1 — Table S1. Characteristics of the nested cohort. (PDF 51 kb) [file 13054_2019_2359_MOESM1_ESM.pdf]

ADDITIONAL TABLE A: Characteristics of the nested cohort study population

	All<br>N = 1324	OHCA<br>Survivors<br>N = 463	OHCA<br>Non-survivors<br>N = 309	IHCA<br>Survivors<br>N = 228	IHCA<br>Non-survivors<br>N = 324
Age (years)	63 (54-71)	60 (50-68)	64 (56-73)	64 (52-71)	66 (58-75)
Gender (male)	73%	77%	77%	67%	67%
Physical status (independent)[1]	91%	97%	92%	92%	82%
ROSC delay (min)[2]	15 (9-22)	17 (12-23)	22 (17-27)	5 (2-10)	10 (4-15)
Shockable[3]	60%	84%	62%	47%	28%
Witnessed[4]	90%	90%	85%	97%	89%
24h-EC-SOFA	7 (6-9)	6 (5-7)	7 (6-8)	7 (6-10)	9 (7-11)
24h-SOFA	9 (7-11)	7 (6-9)	10 (7-11)	9 (7-11)	11 (8-13)
Poor outcome (CPC 3-5)[5]	55%	7.4%	100%	13%	100%
One-year mortality	48%	0%	100%	0%	100%
One-year costs (€1000)	32 (14-63)	48 (30-72)	13 (8.0-24)	62 (32-110)	19 (11-41)
Cost per day alive (€)	400 (130-2500)	130 (83-200)	2500 (1400-3800)	170 (89-310)	2700 (1500-4400)

[1]Simplified WHO/ECOG-classification before cardiac arrest; [2]ROSC delay, time from collapse to return of spontaneous circulation; [3]Shockable, initial cardiac rhythm during resuscitation shockable (ventricular fibrillation/tachycardia); [4]Witnessed, collapse witnessed; [5]Poor outcome, poor neurologic outcome one year after cardiac arrest (CPC 3-5)
