# Supplementary material for: Association of extracerebral organ failure with 1-year survival and healthcare-associated costs after cardiac arrest: an observational database study
Source: Crit Care. 2019 Feb 28;23:67. doi: 10.1186/s13054-019-2359-z (PMC6396453; doi:10.1186/s13054-019-2359-z)
Supplement: Supplementary file 2 — Figure S1. Distribution of EC-SOFA and SOFA score in outcome groups. (PDF 55 kb) [file 13054_2019_2359_MOESM2_ESM.pdf]

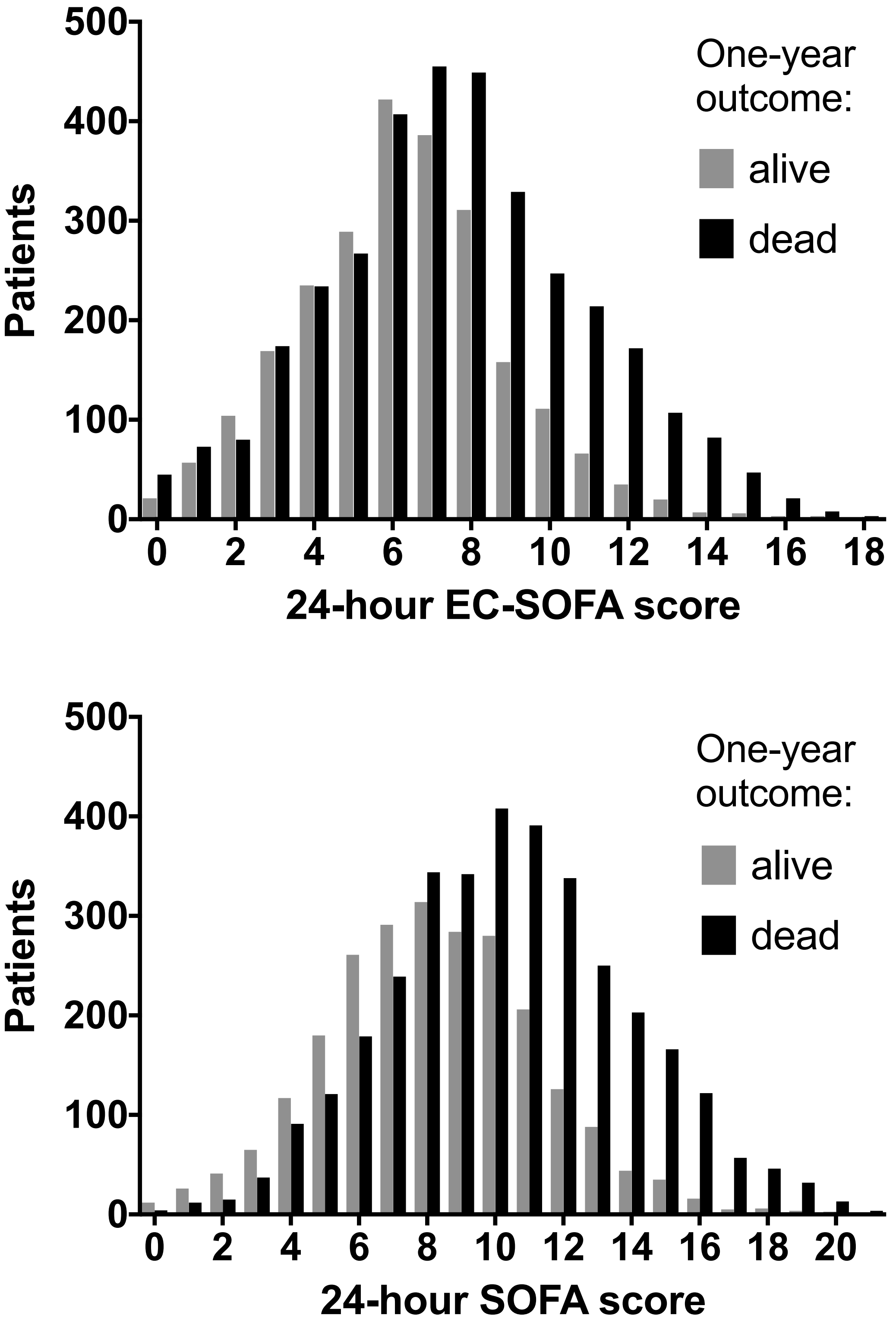

Additional Figure A. Distribution of 24h-EC-SOFA score (upper panel) and 24h-SOFA score (full score including CNS-sub-score; lower panel) in one-year outcome groups. Grey bars, one-year survivors after cardiac arrest; black bars, one-year non-survivors after cardiac arrest.
