# Supplementary material for: Association of extracerebral organ failure with 1-year survival and healthcare-associated costs after cardiac arrest: an observational database study
Source: Crit Care. 2019 Feb 28;23:67. doi: 10.1186/s13054-019-2359-z (PMC6396453; doi:10.1186/s13054-019-2359-z)
Supplement: Supplementary file 3 — Figure S2. One-year survival stratified by admission year. (PDF 38 kb) [file 13054_2019_2359_MOESM3_ESM.pdf]

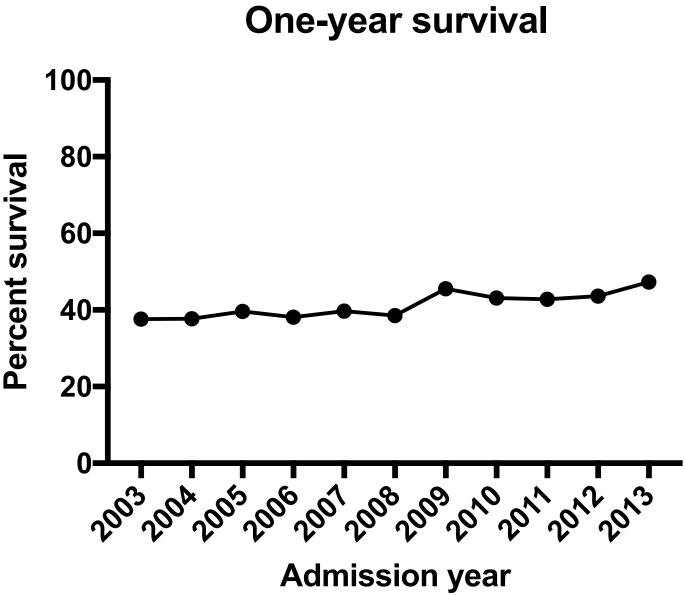

Additional Figure B: One-year survival of cardiac arrest patients stratified by admission year in the full data.
