# Supplementary material for: Association of extracerebral organ failure with 1-year survival and healthcare-associated costs after cardiac arrest: an observational database study
Source: Crit Care. 2019 Feb 28;23:67. doi: 10.1186/s13054-019-2359-z (PMC6396453; doi:10.1186/s13054-019-2359-z)
Supplement: Supplementary file 4 — Table S2. Logistic regression model for the association of the SOFA score with outcome. (PDF 43 kb) [file 13054_2019_2359_MOESM4_ESM.pdf]

ADDITIONAL TABLE B: Logistic regression of the association of the full 24h-SOFA score (including neurologic sub-score) with one-year outcome

	Full Data			Nested cohort					
	One-year mortality			One-year mortality			Poor neurologic outcome[1]		
	OR	95% CI	P	OR	95% CI	P	OR	95% CI	P
Age (year)	1.02	1.01 - 1.02	< 0.01	1.03	1.02 - 1.04	< 0.01	1.03	1.02 - 1.04	< 0.01
Physical status (dependent)[2]	2.54	2.12 - 3.03	< 0.01	2.57	1.49 - 4.45	< 0.01	3.24	1.76 - 5.96	< 0.01
Not shockable[3]				2.72	1.98 - 3.75	< 0.01	2.93	2.10 - 4.08	< 0.01
ROSC (min)[4]				1.05	1.03 - 1.06	< 0.01	1.04	1.03 - 1.06	< 0.01
Not witnessed[5]				1.66	1.15 - 2.63	0.03	1.70	1.05 - 2.77	0.03
24h-SOFA (point)	1.22	1.20 - 1.24	< 0.01	1.22	1.16 - 1.28	< 0.01	1.19	1.14 - 1.25	< 0.01

[1]Cerebral Performance Category (CPC) 3-5 one year after cardiac arrest; [2]Simplified WHO/ECOG-classification before cardiac arrest; [3]Not shockable, initial cardiac rhythm during resuscitation not shockable (asystole/pulseless electrical activity); [4]ROSC delay, time from collapse to return of spontaneous circulation; [5]Not witnessed, collapse not witnessed
