# Supplementary material for: Association of extracerebral organ failure with 1-year survival and healthcare-associated costs after cardiac arrest: an observational database study
Source: Crit Care. 2019 Feb 28;23:67. doi: 10.1186/s13054-019-2359-z (PMC6396453; doi:10.1186/s13054-019-2359-z)
Supplement: Supplementary file 5 — Table S3. Logistic regression model for the association of the EC-SOFA sub-score with outcome in the full data. (PDF 40 kb) [file 13054_2019_2359_MOESM5_ESM.pdf]

ADDITIONAL TABLE C: Logistic regression model of the association of 24h-EC-SOFA sub-score with one-year outcome.

	Full Data		
	One-year mortality		
	OR	95% CI	P
Age (year)	1.01	1.01 - 1.02	< 0.01
Physical status (dependent)[1]	2.16	1.76 - 2.66	< 0.01
Respiration (point)	1.18	1.12 - 1.25	< 0.01
Coagulation (point)	1.09	1.00 - 1.18	0.04
Liver (point)	1.23	1.10 - 1.37	< 0.01
Renal (point)	1.52	1.44 - 1.61	< 0.01

All 24h-EC-SOFA sub-scores (cardiovascular, respiration, coagulation, liver, renal) were considered in a stepwise manner. Only sub-scores with independent predictive value were included in the final model.[1]Simplified WHO/ECOG-classification before cardiac arrest
