# Supplementary material for: Association of extracerebral organ failure with 1-year survival and healthcare-associated costs after cardiac arrest: an observational database study
Source: Crit Care. 2019 Feb 28;23:67. doi: 10.1186/s13054-019-2359-z (PMC6396453; doi:10.1186/s13054-019-2359-z)
Supplement: Supplementary file 6 — Table S4. Univariate analysis of variables used in multivariate models. (PDF 69 kb) [file 13054_2019_2359_MOESM6_ESM.pdf]

ADDITIONAL TABLE D: Univariate analysis of all variables used in the multivariate models (logistic and linear regression) presented in other tables. The values presented in this table are for each variable alone without adjusting for other variables.

	Full Data		
	One-year mortality		
Univariate logistic	OR	95% CI	P
Age (year)	1.02	1.02 - 1.03	< 0.01
Physical status (dependent)	2.89	2.44 - 3.42	< 0.01
24h-EC-SOFA (point)	1.15	1.13 - 1.17	< 0.01
24h-SOFA (point)	1.21	1.19 - 1.23	< 0.01
Cardiovascular (point)	1.04	1.00 - 1.08	0.03
Respiration (point)	1.16	1.11 - 1.21	< 0.01
Coagulation (point)	1.28	1.20 - 1.36	< 0.01
Liver (point)	1.40	1.26 - 1.53	< 0.01
Central nervous system (point)	1.32	1.27 - 1.36	< 0.01
Renal (point)	1.62	1.54 - 1.70	< 0.01

	Nested cohort		
	One-year mortality		
Univariate logistic	OR	95% CI	P
Age (year)	1.02	1.02 - 1.03	< 0.01
Physical status (dependent)	3.19	2.08 - 4.91	< 0.01
24h-EC-SOFA (point)	1.20	1.15 - 1.25	< 0.01
24h-SOFA (point)	1.24	1.19 - 1.28	< 0.01
Cardiovascular (point)	1.08	0.99 - 1.19	< 0.01
Respiration (point)	1.21	1.10 - 1.34	< 0.01
Coagulation (point)	1.35	1.18 - 1.55	< 0.01
Liver (point)	1.37	1.15 - 1.63	< 0.01
Central nervous system (point)	1.25	1.11 - 1.42	< 0.01
Renal (point)	1.61	1.47 - 1.77	< 0.01
Not shockable	3.21	2.53 - 4.07	< 0.01
ROSC-delay (min)	1.02	1.01 - 1.03	< 0.01
Not witnessed	1.83	1.27 - 2.64	< 0.01

	Nested cohort		
	Poor neurologic outcome		
Univariate logistic	OR	95% CI	P
Age (year)	1.03	1.02 - 1.03	< 0.01
Physical status (dependent)	4.04	2.45 - 6.67	< 0.01
24h-EC-SOFA (point)	1.17	1.12 - 1.22	< 0.01
24h-SOFA (point)	1.24	1.19 - 1.29	< 0.01
Cardiovascular (point)	1.03	0.94 - 1.13	0.57
Respiration (point)	1.22	1.10 - 1.35	< 0.01
Coagulation (point)	1.30	1.13 - 1.50	< 0.01
Liver (point)	1.29	1.08 - 1.55	< 0.01
Central nervous system (point)	1.31	1.15 - 1.49	< 0.01
Renal (point)	1.58	1.43 - 1.75	< 0.01
Not shockable	3.36	2.61 - 4.32	< 0.01
ROSC-delay (min)	1.02	1.00 - 1.03	0.02
Not witnessed	2.07	1.38 - 3.10	< 0.01

	Full Data		
	Cost per day alive (€)		
Univariate linear	B	95% CI	P
Age (year)	-1.6	-6.1 - 3.0	0.50
Physical status (dependent)	110	-64 - 290	0.21
24h-EC-SOFA (point)	170	150 - 190	< 0.01
24h-SOFA (point)	170	150 - 180	< 0.01
Cardiovascular (point)	140	97 - 190	< 0.01
Respiration (point)	150	94 - 200	< 0.01
Coagulation (point)	420	355 - 490	< 0.01
Liver (point)	240	140 - 340	< 0.01
Central nervous system (point)	150	110 - 190	< 0.01
Renal (point)	430	390 - 480	< 0.01

	Nested cohort		
	Cost per day alive (€)		
Univariate linear	B	95% CI	P
Age (year)	10	0.40 - 20	0.04
Physical status (dependent)	420	-81 - 920	0.10
24h-EC-SOFA (point)	280	230 - 320	< 0.01
24h-SOFA (point)	240	200 - 270	< 0.01
Cardiovascular (point)	200	87 - 320	< 0.01
Respiration (point)	240	121 - 370	< 0.01
Coagulation (point)	560	400 - 720	< 0.01
Liver (point)	410	200 - 610	< 0.01
Central nervous system (point)	110	-51 - 260	0.18
Renal (point)	670	570 - 760	< 0.01
Not shockable	1000	730 - 1300	< 0.01
ROSC-delay (min)	22	10 - 33	< 0.01
Not witnessed	210	-250 - 670	0.38

	Full Data		
	Total costs in one-year survivors (1000€)		
Univariate linear	B	95% CI	P
Age (year)	-0.74	-0.95 - -0.54	< 0.01
Physical status (dependent)	11	0.90 - 22	0.03
24h-EC-SOFA (point)	4.2	3.1 - 5.2	< 0.01
24h-SOFA (point)	3.2	2.3 - 4.1	< 0.01
Cardiovascular (point)	3.4	1.3 - 5.6	< 0.01
Respiration (point)	7.4	5.0 - 9.9	< 0.01
Coagulation (point)	5.4	1.8 - 9.1	< 0.01
Liver (point)	5.4	-0.29 - 11	0.06
Central nervous system (point)	1.9	-0.04 - 3.8	0.06
Renal (point)	8.3	5.5 - 11	< 0.01

	Nested cohort		
	Total costs in one-year survivors (1000€)		
Univariate linear	B	95% CI	P
Age (year)	-0.28	-0.62 - 0.058	0.10
Physical status (dependent)	22	-1.6 - 45	0.07
24h-EC-SOFA (point)	7.1	5.2 - 9.0	< 0.01
24h-SOFA (point)	4.2	2.5 - 5.8	< 0.01
Cardiovascular (point)	1.0	-3.2 - 5.2	0.48
Respiration (point)	6.6	2.4 - 11	< 0.01
Coagulation (point)	11	4.7 - 18	< 0.01
Liver (point)	17	7.9 - 25	< 0.01
Central nervous system (point)	-4.4	-9.7 - 0.98	0.11
Renal (point)	20	15 - 24	< 0.01
Not shockable	21	9.9 - 32	< 0.01
ROSC-delay (min)	-0.19	-0.66 - 0.28	0.43
Not witnessed	-23	-41 - -4.8	< 0.01
