# Supplementary material for: Association of extracerebral organ failure with 1-year survival and healthcare-associated costs after cardiac arrest: an observational database study
Source: Crit Care. 2019 Feb 28;23:67. doi: 10.1186/s13054-019-2359-z (PMC6396453; doi:10.1186/s13054-019-2359-z)
Supplement: Supplementary file 7 — Figure S3. ECPS stratified by admission year. (PDF 40 kb) [file 13054_2019_2359_MOESM7_ESM.pdf]

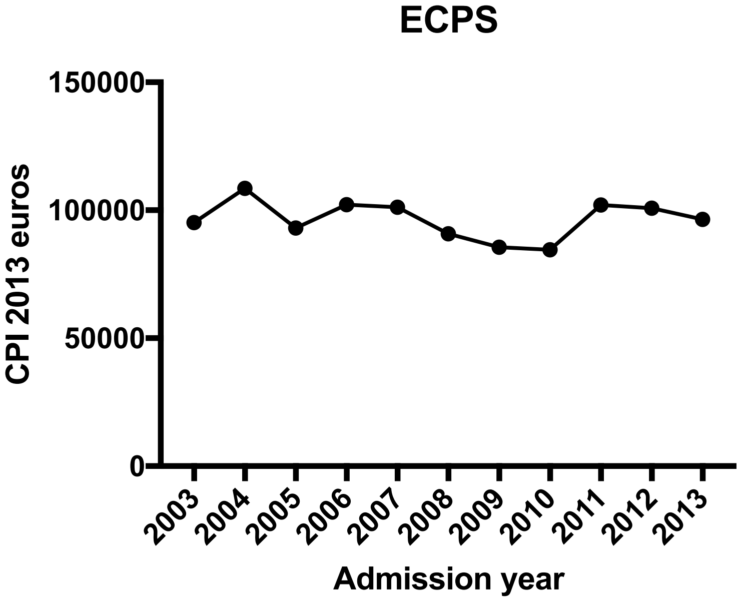

Additional Figure C: Effective cost per survivor (ECPS) stratified by admission year in the full data. ECPS was calculated by dividing the sum of total costs for all patients with the number of survivors for each year.
