# Supplementary material for: Association of extracerebral organ failure with 1-year survival and healthcare-associated costs after cardiac arrest: an observational database study
Source: Crit Care. 2019 Feb 28;23:67. doi: 10.1186/s13054-019-2359-z (PMC6396453; doi:10.1186/s13054-019-2359-z)
Supplement: Supplementary file 8 — Figure S4. Total costs per patient stratified by admission year. (PDF 46 kb) [file 13054_2019_2359_MOESM8_ESM.pdf]

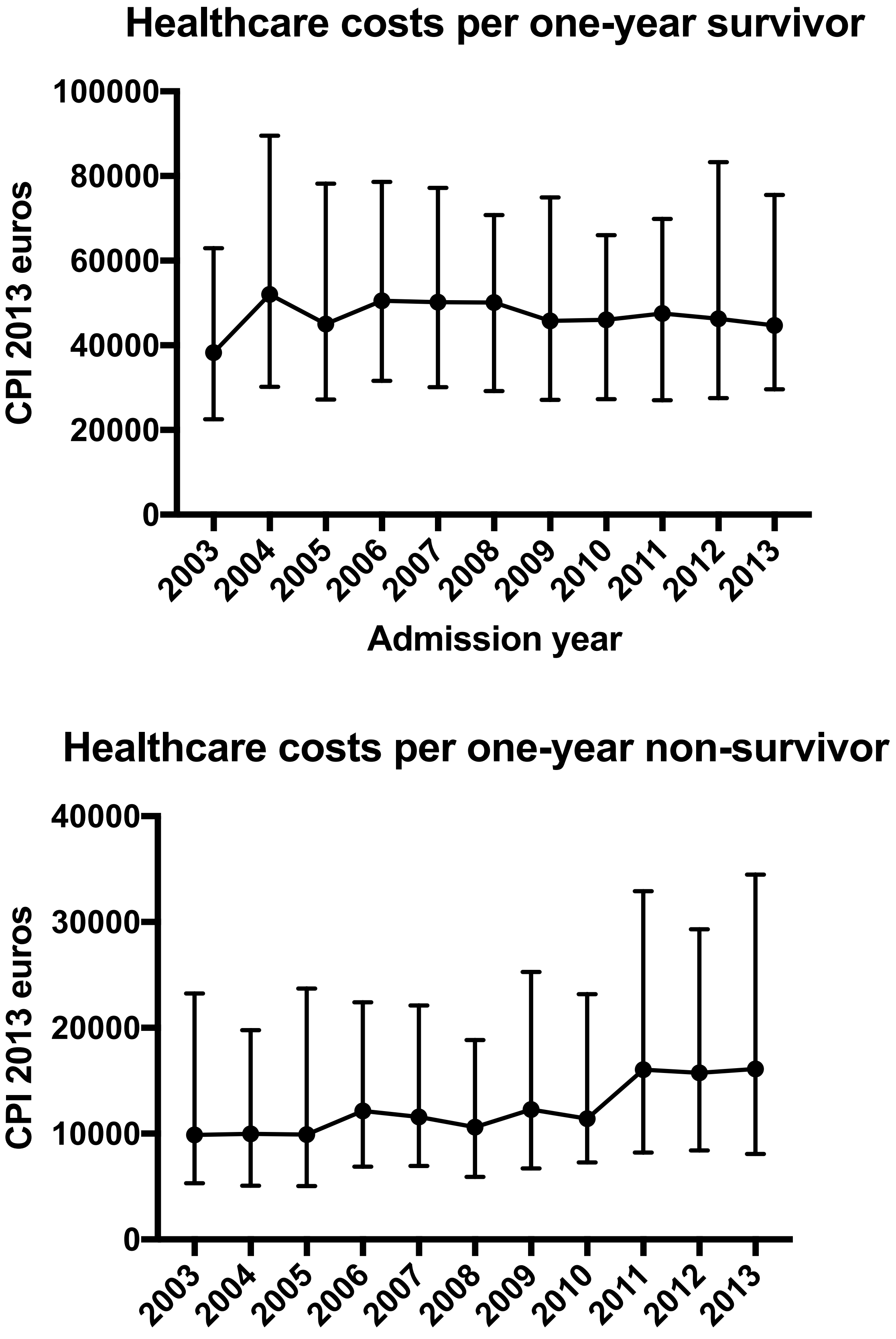

Additional Figure D: Total healthcare-associated costs recorded during the year after cardiac arrest stratified by admission year in the full data. Upper panel, one-year survivors; lower panel, one-year non-survivors. Median with interquartile ratio (IQR, whiskers) is presented.
