# Supplementary material for: Association of extracerebral organ failure with 1-year survival and healthcare-associated costs after cardiac arrest: an observational database study
Source: Crit Care. 2019 Feb 28;23:67. doi: 10.1186/s13054-019-2359-z (PMC6396453; doi:10.1186/s13054-019-2359-z)
Supplement: Supplementary file 9 — Table S5. Logistic regression model for the association of the EC-SOFA sub-score with outcome in the nested cohort. (PDF 40 kb) [file 13054_2019_2359_MOESM9_ESM.pdf]

ADDITIONAL Table E: Linear model of the association of 24h-EC-SOFA sub-score with one-year healthcare-associated costs per day alive.

	Cost per day alive (€)		
	Full data		
	B	95% CI	P
Age (year)	-4.8	-9.2 - -0.34	0.04
Physical status (dependent)[1]	-43	-210 - 130	0.63
Respiration (point)	120	61 - 170	< 0.01
Coagulation (point)	300	230 - 370	< 0.01
Cardiovascular (point)	90	41 - 140	< 0.01
Renal (point)	400	350 - 440	< 0.01

All 24h-EC-SOFA sub-scores (cardiovascular, respiration, coagulation, liver, renal) were considered in a stepwise manner. Only sub-scores with independent predictive value were included in the final model. [1]Simplified WHO/ECOG-classification before cardiac arrest
