# Supplementary material for: Association of extracerebral organ failure with 1-year survival and healthcare-associated costs after cardiac arrest: an observational database study
Source: Crit Care. 2019 Feb 28;23:67. doi: 10.1186/s13054-019-2359-z (PMC6396453; doi:10.1186/s13054-019-2359-z)
Supplement: Supplementary file 10 — Table S6. Logistic regression model for the association of the EC-SOFA score with outcome in OHCA and IHCA sub-groups of the nested cohort. (PDF 40 kb) [file 13054_2019_2359_MOESM10_ESM.pdf]

ADDITIONAL Table F: Linear model of the association of 24h-EC-SOFA sub-score with one-year healthcare-associated costs (per 1000€) in one-year survivors.

	Total costs in one-year survivors (1000€)		
	Full data		
	B	95% CI	P
Age (year)	-0.88	-1.1 - -0.67	< 0.01
Physical status (dependent)[1]	13	2.8 - 24	0.01
Respiration (point)	7.3	4.8 - 9.8	< 0.01
Coagulation (point)	4.6	1.0 - 8.3	0.01
Renal (point)	7.9	5.0 - 11	< 0.01

All 24h-EC-SOFA sub-scores (cardiovascular, respiration, coagulation, liver, renal) were considered in a stepwise manner. Only sub-scores with independent predictive value were included in the final model. [1]Simplified WHO/ECOG-classification before cardiac arrest
