# Supplementary material for: Association of extracerebral organ failure with 1-year survival and healthcare-associated costs after cardiac arrest: an observational database study
Source: Crit Care. 2019 Feb 28;23:67. doi: 10.1186/s13054-019-2359-z (PMC6396453; doi:10.1186/s13054-019-2359-z)
Supplement: Supplementary file 11 — Table S7. Linear regression model for the association of the EC-SOFA sub-score with cost per day alive. (PDF 43 kb) [file 13054_2019_2359_MOESM11_ESM.pdf]

ADDITIONAL TABLE G: Logistic regression model of the association of 24h-EC-SOFA sub-score with one-year outcome in the nested cohort.

	Nested cohort									
	One-year mortality					Poor neurologic outcome[1]				
	OR	95% CI			P	OR	95% CI			P
Age (year)	1.03	1.02	-	1.04	< 0.01	1.03	1.02	-	1.04	< 0.01
Physical status (dependent)[2]	2.39	1.38	-	4.14	< 0.01	3.27	1.75	-	6.11	< 0.01
Not shockable[3]	3.09	2.26	-	4.22	< 0.01	3.41	2.46	-	4.74	< 0.01
ROSC delay (min)[4]	1.05	1.03	-	1.07	< 0.01	1.05	1.03	-	1.07	< 0.01
Not witnessed[5]	1.79	1.14	-	2.82	0.01	2.00	1.22	-	3.26	< 0.01
Respiration (point)	1.16	1.02	-	1.32	0.03	1.17	1.02	-	1.33	0.02
Renal (point)	1.50	1.32	-	1.70	< 0.01	1.42	1.25	-	1.63	< 0.01

All 24h-EC-SOFA sub-scores (cardiovascular, respiration, coagulation, liver, renal) were considered in a stepwise manner. Only sub-scores with independent predictive value were included in the final model. [1]Cerebral Performance Category (CPC) 3-5 one year after cardiac arrest; [2]Simplified WHO/ECOG-classification before cardiac arrest; [3]Not shockable, initial cardiac rhythm during resuscitation not shockable (asystole/pulseless electrical activity); [4]ROSC delay, time from collapse to return of spontaneous circulation; [5]Not witnessed, collapse not witnessed
