# Supplementary material for: Association of extracerebral organ failure with 1-year survival and healthcare-associated costs after cardiac arrest: an observational database study
Source: Crit Care. 2019 Feb 28;23:67. doi: 10.1186/s13054-019-2359-z (PMC6396453; doi:10.1186/s13054-019-2359-z)
Supplement: Supplementary file 12 — Table S8. Linear regression model for the association of the EC-SOFA sub-score with total costs in 1-year survivors. (PDF 46 kb) [file 13054_2019_2359_MOESM12_ESM.pdf]

ADDITIONAL TABLE H: Logistic regression model of the association of 24h-EC-SOFA score with one-year outcome in the out-of-hospital cardiac arrest (OHCA) and in-hospital cardiac arrest (IHCA) subgroups of the nested cohort.

	OHCA					
	One-year mortality			Poor neurologic outcome[1]		
	OR	95% CI	P	OR	95% CI	P
Age (year)	1.04	1.02 - 1.05	< 0.01	1.04	1.02 - 1.05	< 0.01
Physical status (dependent)[2]	2.23	0.97 - 5.13	0.06	3.36	1.32 - 8.55	0.01
Not shockable[3]	4.26	2.80 - 6.50	< 0.01	4.67	2.98 - 7.30	< 0.01
ROSC delay (min)[4]	1.06	1.04 - 1.08	< 0.01	1.07	1.04 - 1.09	< 0.01
Not witnessed[5]	1.43	0.84 - 2.45	0.19	1.61	0.92 - 2.83	0.10
24h-EC-SOFA (point)	1.20	1.10 - 1.31	< 0.01	1.15	1.05 - 1.25	< 0.01

[1]Cerebral Performance Category (CPC) 3-5 one year after cardiac arrest; [2]Simplified WHO/ECOG-classification before cardiac arrest; [3]Not shockable, initial cardiac rhythm during resuscitation not shockable (asystole/pulseless electrical activity); [4]ROSC delay, time from collapse to return of spontaneous circulation; [5]Not witnessed, collapse not witnessed

	IHCA					
	One-year mortality			Poor neurologic outcome[1]		
	OR	95% CI	P	OR	95% CI	P
Age (year)	1.02	1.00 - 1.04	0.02	1.02	1.00 - 1.04	0.07
Physical status (dependent)[2]	2.84	1.37 - 5.89	< 0.01	3.50	1.52 - 8.04	< 0.01
Not shockable[3]	2.58	1.57 - 4.24	< 0.01	2.79	1.66 - 4.68	< 0.01
ROSC delay (min)[4]	1.03	1.00 - 1.06	0.04	1.03	1.00 - 1.06	0.08
Not witnessed[5]	4.65	1.63 - 13.24	< 0.01	5.65	1.59 - 20.02	< 0.01
24h-EC-SOFA	1.15	1.06 - 1.24	< 0.01	1.11	1.03 - 1.21	< 0.01
