# Supplementary material for: Association of extracerebral organ failure with 1-year survival and healthcare-associated costs after cardiac arrest: an observational database study
Source: Crit Care. 2019 Feb 28;23:67. doi: 10.1186/s13054-019-2359-z (PMC6396453; doi:10.1186/s13054-019-2359-z)
Supplement: Supplementary file 13 — Table S9. Linear regression model for the association of the EC-SOFA sub-score with cost per day alive in OHCA and IHCA sub-groups of the nested cohort. (PDF 43 kb) [file 13054_2019_2359_MOESM13_ESM.pdf]

ADDITIONAL Table I: Linear model of the association of 24h-EC-SOFA score with one-year healthcare associated costs per day alive in the out-of-hospital cardiac arrest (OHCA) and in-hospital cardiac arrest (IHCA) subgroups of the nested cohort.

	Cost per day alive (€)					
	OHCA			IHCA		
	B	95% CI	P	B	95% CI	P
Age (year)	12	1.6 - 23	0.02	-9.1	-26 - 8.1	0.30
Physical status (dependent)[1]	-15	-690 - 660	0.97	300	-370 - 980	0.38
Not shockable[2]	1000	670 - 1300	< 0.01	410	-94 - 910	0.11
ROSC delay (min)[3]	38	24 - 53	< 0.01	47	18 - 75	< 0.01
Not witnessed[4]	32	-410 - 480	0.89	530	-310 - 1400	0.21
24h-EC-SOFA (point)	210	150 - 280	< 0.01	200	120 - 270	< 0.01

[1]Simplified WHO/ECOG-classification before cardiac arrest; [2]Not shockable, initial cardiac rhythm during resuscitation not shockable (asystole/pulseless electrical activity); [3]ROSC delay, time from collapse to return of spontaneous circulation; [4]Not witnessed, collapse not witnessed
