# Supplementary material for: Association of extracerebral organ failure with 1-year survival and healthcare-associated costs after cardiac arrest: an observational database study
Source: Crit Care. 2019 Feb 28;23:67. doi: 10.1186/s13054-019-2359-z (PMC6396453; doi:10.1186/s13054-019-2359-z)
Supplement: Supplementary file 14 — Table S10. Linear regression model for the association of the EC-SOFA sub-score with total costs in 1-year survivors in OHCA and IHCA sub-groups of the nested cohort. (PDF 43 kb) [file 13054_2019_2359_MOESM14_ESM.pdf]

ADDITIONAL Table J: Linear model of the association of 24h-EC-SOFA score with one-year healthcare associated costs (per 1000€) in one-year survivors in the out-of-hospital cardiac arrest (OHCA) and in-hospital cardiac arrest (IHCA) subgroups of the nested cohort.

	Total costs in one-year survivors (1000€)									
	OHCA					IHCA				
	B	95% CI			P	B	95% CI			P
Age (year)	-0.093	-0.42	-	0.24	0.58	-1.3	-2.3	-	-0.31	0.01
Physical status (dependent)[1]	-7.4	-35	-	20	0.60	63	11	-	110	0.02
Not shockable[2]	7.8	-4.5	-	20	0.22	16	-13	-	44	0.28
ROSC delay (min)[3]	0.37	-0.10	-	0.84	0.12	.39	-1.2	-	1.9	0.62
Not witnessed[4]	-13	-28	-	2.5	0.10	-69	-150	-	11	0.09
24h-EC-SOFA (point)	2.5	0.20	-	4.8	0.03	11	5.6	-	16	< 0.01

[1]Simplified WHO/ECOG-classification before cardiac arrest; [2]Not shockable, initial cardiac rhythm during resuscitation not shockable (asystole/pulseless electrical activity); [3]ROSC delay, time from collapse to return of spontaneous circulation; [4]Not witnessed, collapse not witnessed
